# Sporadic Hepatocellular Carcinoma in an Adolescent Harboring a Somatic CDH1 Mutation: A Case Report and Literature Review

**DOI:** 10.1002/kjm2.70236

**Published:** 2026-05-07

**Authors:** Chang‐Wei Su, Peir‐In Liang, Shih‐Chang Chuang

**Affiliations:** ^1^ Division of General and Digestive Surgery, Department of Surgery Kaohsiung Medical University Hospital Kaohsiung Taiwan; ^2^ Department of Pathology Kaohsiung Medical University Hospital Kaohsiung Taiwan; ^3^ Faculty of Medicine, College of Medicine, Kaohsiung Medical University Kaohsiung Taiwan

1

Primary liver malignancy is rare in children, and hepatoblastoma and hepatocellular carcinoma (HCC) are the most common variants. Unlike adult HCC, pediatric HCC is more frequently sporadic or associated with congenital metabolic disorders. Surgical resection offers the only curative potential, but many patients present with unresectable, advanced disease. Unfortunately, advanced cases have a five‐year survival rate of less than 30% [1]. Understanding the molecular landscape is crucial for improving outcomes. While mutations in TERT, CTNNB1, and TP53 are well‐characterized in adult HCC [2], the genetic drivers in pediatric sporadic HCC remain poorly understood. Herein, we present a rare case of sporadic HCC in a healthy adolescent involving a CDH1 mutation identified via comprehensive genomic profiling.

A 17‐year‐old male with no significant past medical history presented to our outpatient department for evaluation of an asymptomatic, mildly enlarged, segment 6/7 liver tumor. Laboratory results were within normal limits, including alpha‐fetoprotein (AFP: 0.9 ng/mL) and hepatitis viral screenings (HBsAg(−), anti‐HBs(+), IgG anti‐HBc(−), anti‐HCV(−)). Initial computed tomography images revealed a 2.8 cm tumor in segments 6/7 of the liver. He underwent a biopsy, which revealed “atypical hepatocellular carcinoma.” Fearing pain, the patient refused additional sampling. We scheduled regular follow‐up appointments with tumor marker surveillance every 3 months and abdominal echography every 6 months. However, he was lost to follow‐up.

Four years later, the patient underwent magnetic resonance imaging that revealed tumor growth to 3.4 cm and indicated early arterial phase enhancement and regression (Figure 1A,B). AFP levels were within normal range during follow‐up. We performed a laparoscopic partial hepatectomy of segment 6/7, and the pathology examination revealed HCC, grade 1, pT1bNx. Microscopically, the tumor demonstrated thickened cell plates with a trabecular pattern and increased reticulin on silver staining. Immunohistochemistry staining was positive for glutamine synthetase (diffuse/strong) and CD34 (indicating a diffuse capillary network), and negative for CK7, confirming hepatocellular origin without biliary features (Figure 1C,D).

**FIGURE 1:**
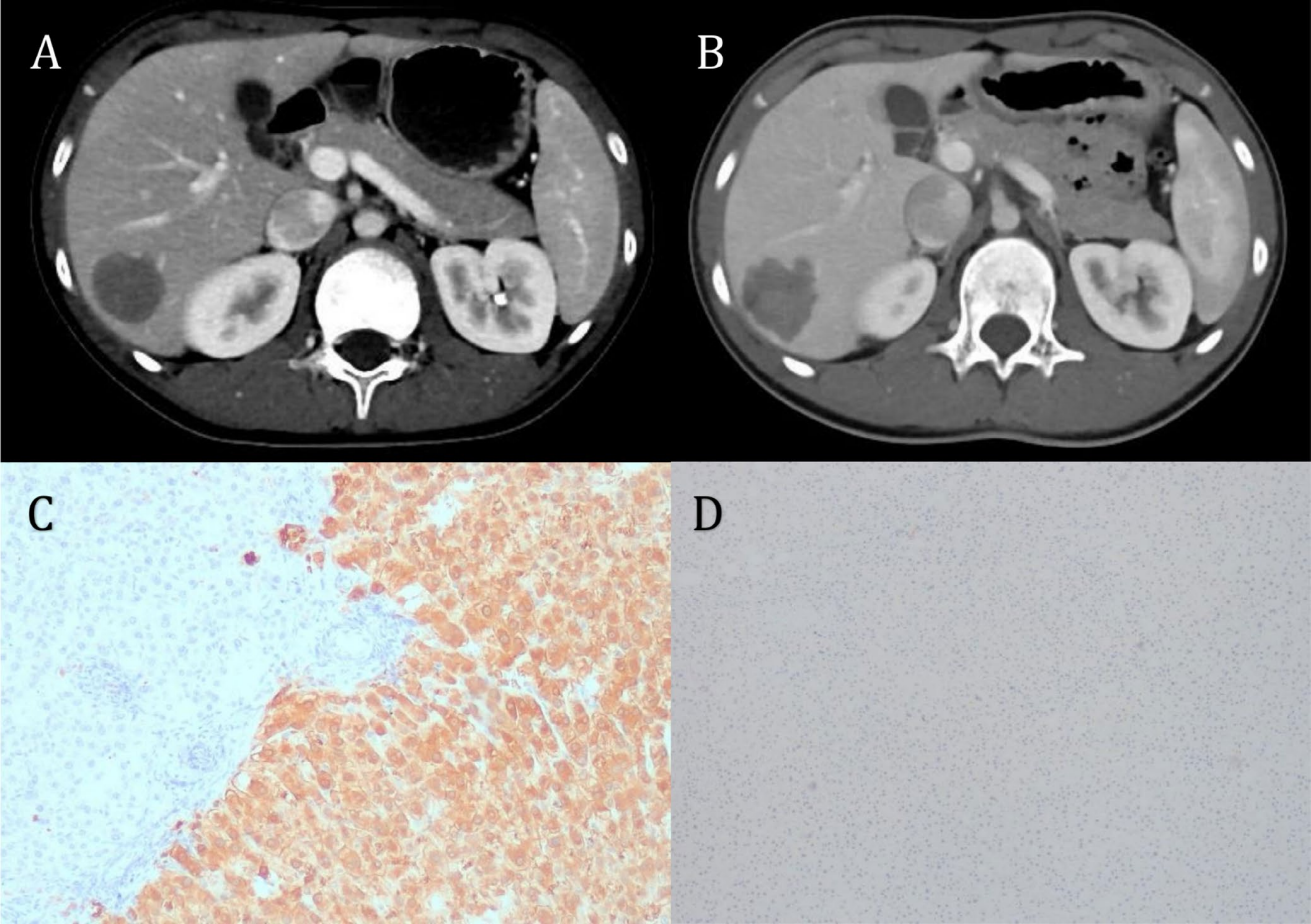
Contrast‐enhanced abdomen CT at initial presentation (A) and 4 years later (B) revealed mild enlargement of the segment 6/7 liver tumor (2.8 cm to 3.4 cm) with a lobulated margin and poor enhancement phase. The tumor demonstrated strongly positive glutamine synthetase staining (C). Additionally, CK7 staining (D) revealed that the tumor had no obvious bile duct.

To assess prognosis and potential therapeutic targets, a positron emission tomography scan and next‐generation sequencing (NGS) (ACTOnco+, covering 440 cancer‐related genes) were performed. The NGS analysis identified a pathogenic CDH1 frameshift mutation, R796fs (c.2386dup), with a 26.7% allele frequency (VAF). Given the absence of residual disease and the lack of targeted therapies for CDH1 loss‐of‐function, the patient is currently undergoing regular imaging and tumor marker surveillance.

Pediatric HCC can be classified as either sporadic, arising in a normal liver, or secondary, arising from underlying liver disease [3]. Large‐scale HCC genomic studies have identified the TERT promoter (37%–59%), TP53 (30%–55%), CTNNB1 (27%–35%), and AXIN1 (5%–15%) as the most frequent driver mutations [2, 4]. They are involved in the disruption of telomere maintenance, cell cycle regulation, the Wnt/beta‐catenin pathway, and epigenetic modification, respectively [2]. Our patient lacked these classical mutations; instead, NGS revealed a frameshift mutation in CDH1 (R796fs).

CDH1 encodes E‐cadherin, a transmembrane glycoprotein crucial for cell–cell adhesion and tissue polarity [5]. Loss of E‐cadherin is a hallmark of the epithelial‐mesenchymal transition (EMT), a process associated with tumor invasion and metastasis, and possible epithelial tumor development [5]. One study reported that EMT may occur via CDH1 promoter hypermethylation in HCC, with the frequency varying from 13.3% to 66.7% [5]. This inspired our team to consider whether a CDH1‐related mutation or modification may induce HCC development. Germline CDH1 mutations are causally linked to hereditary diffuse gastric cancer and lobular breast cancer. However, in this case, the 26.7% allele frequency in a sample with 70% tumor purity strongly suggests that this is a somatic heterozygous mutation rather than a germline defect.

While CDH1 mutations are rare in HCC, downregulation of E‐cadherin via CDH1 promoter hypermethylation is frequently observed and correlates with metastasis and poor prognosis [5]. Our patient's specific mutation, R796fs, causes a premature truncation of the protein, likely leading to loss of function. Although rare, this finding aligns with the hypothesis that disruption of the E‐cadherin pathway, whether by methylation or mutation, contributes to hepatocarcinogenesis.

We presented an uncommon case of indolent, sporadic HCC in a healthy adolescent. The integration of genomic profiling identified a somatic CDH1 mutation, expanding the known pediatric HCC molecular mechanism beyond the classical drivers. This case underscores the importance of molecular testing in pediatric liver tumors to better understand their pathogenesis and guide future precision medicine strategies.

## Conflicts of Interest

The authors declare no conflicts of interest.

## Data Availability

The data that support the findings of this study are available from the corresponding author upon reasonable request.
